# Some Surgical Cases*Read at Beaumont meeting South Texas District Medical Association, August, 1900. Report of Chairman of Surgical Section.

**Published:** 1900-09

**Authors:** J. S. Price

**Affiliations:** Beaumont, Texas


### TEXAS MEDICAL JOURNAL.

ESTABLISHED JULY, 1885.

PUBLISHED MONTHLY.—SUBSCRIPTION $1.00 A YEAR.

VOL. XVI. AUSTIN, SEPTEMBER, 1900. No. 3.

### Original Contributions.

For the Texas Medical Journal.

### Some Surgical Cases.*

J. S. PRICE, M. D., BEAUMONT, TEXAS.

Instead of consuming your time by going over the recent advances in surgery, I shall make a slight departure, by relating a few cases coming under my care during the past year or two, which I trust will be of at least passing interest.

The first case to which I shall call your attention was

1. CARCINOMA OF THE UTERUS

in an advanced stage, under the care of my friend and colleague, Dr. B. F. Haynes, of this city. The true state of affairs was not suspected until the case came into the hands of Dr. Haynes, who recognized the condition, and had me see her with him. While we rather doubted that operative measures would be of more benefit than to relieve the exsanguinating effects of hemorrhage, we advised an operation, to which she readily consented. Knowing that the uterus was firmly fixed, and that the cervix was partially destroyed, we determined on the use of Bernays' utero-tractor, with which possibly most of you are familiar. The advantages claimed for this instrument, I think, are well grounded, especially in cases of ad-

*Read at Beaumont meeting South Texas District Medical Association, August, 1900. Report of Chairman of Surgical Section.

vanced cancer where the tissues are friable and break away easily from the bite of a vulsellum. Dr. Bernays claims: First—That it takes away no space in the vault of the vagina surrounding the cervix, since it seizes the womb from within the cavity; when closed, the scissor-like handles approach one another so that they occupy very little space either within or without the vagina. Second—The instrument can be introduced into the cervical canal or the womb cavity any distance desired; whatever amount of traction may be brought to bear upon the tissues of the womb, a corresponding resistability can be created by opening the branches of the tractor more or less. Third—The four tooth-like prongs of each branch are not the only factors that resist tearing out; the wedge shape of the open branches also forms an important obstacle tending to prevent this accident. A glance at the cuts will show the point of the wedge situated in or near the cervix, while the broad end of the wedge looks towards the fundus. Fourth—the utero-tractor can be held either in the left hand of the operator, or can be entrusted to an assistant. A great advantage in the use of the instrument lies in the circumstance that traction is not solely made upon the vaginal portion by the vulsellum or Musseaux's forceps, or by the loop of thread heretofore in use. The instrument has its points of attachment upon the whole length of the uterus, if desired. We are thus enabled to move the organ in toto, from right to left, and can put the opposite parametrium tissue, yes, the whole of the broad ligament, on the stretch. This is of considerable importance, and will, no doubt, be appreciated by those operators who tie the bleeding points in the womb as they proceed with the extirpation. Fifth—The tractor can be introduced as easily as the uterine probe, and also can be removed at any time with but little trouble. It may also be used to facilitate the tipping over or doubling up of the womb upon itself, through the anterior or posterior cul-de-sac. The laceration of the organ, which must be caused by the use of the instrument is of no importance, as it is intended only for those operations which propose the total removal of the womb. The instrument also obviates the sometimes disagreeable sponging necessitated by the vulsellum or other forceps used for dragging down the womb. A little absorbent cotton used around the joint of the blade stops all bleeding if there be any from within the womb.

We trust you will pardon this somewhat prolix description of this instrument, yet we feel safe in predicting that you will be pleased with it after giving it a trial, and for this reason the case was called to your attention. The uterus, though firmly fixed, was removed with very little difficulty, and the getting well was uneventful. As a matter of course, we anticipated a recurrence of the disease, yet we have reasons for congratulations, as we gave her two years of relief. Up to three months ago she felt perfectly well, but I am told that the inevitable has happened, and that she is doomed now to but a few more months of suffering.

### 2. DEPRESSED FRACTURE OVER PARIETO-FRONTAL BONES.

Mr. J. S. H., a real estate agent, while out with a party of friends inspecting rice lands, stopped his team for lunch, and while thus engaged his hat was blown off his head and fell close to one of his mule's heels. In the act of picking it up, he was kicked by one of the animals on the right side of the head. He was immediately unconscious, and so remained for several hours. His companions washed the wound, succeeded in getting him into the vehicle, and brought him to town. I was called, and upon examination, a wound about two inches in length and about three-quarters of an inch in width was found on the right side of the head, involving chiefly the parietal, and extending forward on the frontal bone about half an inch; fragments depressed and comminuted. Several hours had elapsed, and when seen he was in a semi-conscious state; his eyes were set as if he were on the verge of a convulsive seizure. There was no disturbance of locomotion, and he complained very little when aroused and questioned. When told the condition he was in, he absolutely refused to have anything done further than the closing of the wound, stating he was determined to go to his home in Kansas City the next morning. Finding argument of no avail, the wound was thoroughly cleansed of the dirt and hair. His friends had tried to close the wound with postage stamps. Upon leaving him, I advised his friends that if he changed his mind during the night, I stood ready to serve him. About three o'clock next morning he aroused, called one of his friends, who related to him what had transpired, and he professed absolute ignorance of everything that had occurred after the accident, and had me called at once, as he was anxious for the operation. He was taken to the hospital next morning, and under the usual technique, the fragments of bone were removed without any trouble. Dr. Goeber gave the anaesthetic. It was discovered that the patient, on being placed on the table, was still suffering extremely from the shock, pulse from 140 to 150, and at times almost imperceptible. We had previously learned of his being a constant drinker, and you well know how this class of patients stand chloroform. Hence, we decided to give him a minimum of chloroform and a maximum of morphine and whiskey, after Professor Wyeth's suggestion. Though he drank the whiskey with considerable reluctance, we were enabled to proceed and complete our work, where under chloroform or ether, I believe his life would have paid the penalty. The case made an uneventful recovery.

### 3. UNUNITED FRACTURE OF FEMUR.

In November of last year, a young man of about twenty, lost control of his team of mules, one of his lines having broken while he was hauling a heavy load of rice. He was precipitated to the ground, under the wheels of his wagon, which passed over his left thigh, fracturing it in an oblique direction at the junction of the upper with the middle third. These were the facts elicited from the courier who came after me. Dr. Nash, of this city, kindly consented to go with me, and we reached him about nine o'clock at night. Splints were improvised and temporary dressing applied looking forward to placing the limb in permanent dressing later. Our advice was to have him brought into the city, to which all agreed. An ambulance was sent next day, but after more mature deliberation they decided against us, and here is where our trouble began. After one week, primary dressing was removed, and plaster applied. The deformity recurred as soon as dressing was removed, and the profoundest anaesthesia had to be introduced in order to overcome muscular spasms. During the first seven weeks he suffered almost continually with malarial fever, which would yield to quinine for a short while, and then recur. Thus is was we were kept on the jump with couriers coming and going. At last, after seven weeks of mental anguish to all parties concerned, the plaster was removed, and the patient was turned over in bed. Dr. Henderson, who removed the dressing, reported everything as being satisfactory, union seemed complete, but, alas! our troubles had only begun, as union was imperfect. About a week after the removal of the dressing, the young man's father came in and announced that the patient was unable to move the limb, and that the old deformity had recurred. We went again, prepared to apply another plaster bandage, knowing full well that we had to deal with an ununited fracture, which proved to be the case. He was again anaesthetized, the ends of the bone well rubbed together and again placed in plaster. We did not remove the last splint for two months, during which time his general health improved wonderfully. We are glad to report that the final results are all that could be expected, perfect union with very little shortening.

4. SUPPURATIVE APPENDICITIS AS A SEQUEL TO SCARLET FEVER.

During the epidemic of scarlet fever, from which all sections of Texas suffered, Beaumont contributed her proportion of cases. Among the interesting sequelæ, I shall narrate a case of suppurative appendicitis, with rupture into the peritoneal cavity. The case in question was under the care of my friend, Dr. Blewitt, of this city, who kindly asked me to attend it with him. Several members of the family were stricken with the fever, one after the other, three being confined to the bed at one time, and if I am correct in my recollection, the subject of these notes was the fourth to come down with the fever. All of these cases had the typical rash, sore throat, enlarged glands, and finally disquamated. The young boy at about the fourth or fifth day of his fever, which was running from 101 to 102, developed an excruciating pain over McBurbey's point. Associated with the pain there was an exacerbation of temperature, which ran as high as 104; abdominal rigidity, gastric disturbance, inability to rest without an opiate. The parents were advised of the turn of affairs and consented to an operation, which we did three days after the first warning symptoms. Upon entering the abdomen there was a rush of foul-smelling pus. We spent no time in searching for the appendix, believing that it had been completely destroyed in the inflammatory process, but contented ourselves with cleansing the intestines of pus by mopping thoroughly with sterilized gauze. The wound was partially closed, with space left for wicks of iodoform gauze, which expedited drainage beautifully. The temperature next morning was 99, pulse normal, and the boy had no further untoward symptoms.

This case should be remembered, resulting along with scarlet fever. Whether we shall class it as a sequence is for you to say.

The following points, though not new, are intended to be brought out in the report of the above cases:

First—In vaginal hysterectomy, where the uterus is firmly fixed, you will find Bernays' utero-tractor of great value.

Second—In those cases where chloroform or ether is contra-indicated, we have a safe recourse in whiskey and morphine with a minimum of either of the anaesthetics.

Third—In cases of fracture of the large bones, where there is a condition of impoverished health, we should delay the removal of our dressing. Because the present age is one of operative enthusiasm, and because we are now able to operate with greater safety than formerly, is no reason why every one should follow the example of those most advanced in operative ability. Conservatism is, too, advised.

Finally, among other sequelæ of scarlet fever, we should be on the alert for appendicitis. It is very likely to result from the catarrhal disturbances of the gastro-intestinal tract.

